# Anatomy, Physiology and Hygiene

**Published:** 1885-05

**Authors:** 


**Anatomy, Physiology and Hygiene.** A Manual for the use of Schools, Colleges and the General Readers. By JEROME WALKER, M. D., Lecturer on Anatomy, Physiology and Hygiene in the Central School, Brooklyn, and on Diseases of Children in the Long Island College Hospital; Senior Physician to the Seaside Home for Children, Coney Island, etc. New York: A. Lorill & Co., No. 16 Astor Place. 1884.

We have of late received many books on this subject, and while they have all contained much to commend, all have been more or less faulty. The work before us is a decided improvement upon any of its predecessors. This is not a book which teaches a little smattering of anatomy and physiology and leaves the scholar ignorant of the application of that knowledge to the preservation of health. Dr. Walker teaches the essential facts of anatomy and physiology and makes a constant application of physiological principles to hygienic ends. The *Journal of Education* says of it that in clearness of statement, exactness of description, fullness of illustration, and in its practical application to daily life, it has not been excelled if equaled by any other American author. This is not exaggerated praise. Medical men can recommend the book with confidence.

